# Round the Hospitals

**Published:** 1919-10-25

**Authors:** 


### ROUND THE HOSPITALS.

On October 18, by command of the King, Lieut.-Colonel Prince Arthur of Connaught held an Investiture in the Throne Room, St. James's Palace, at 11.30, on his Majesty's behalf. The following decorations were bestowed on members of the Nursing Services: Royal Red Cross, First Class, Matron Lily Ephgrave, Q.A.I.M.N.S., and Miss Elizabeth Orr, Q.A.I.M.N.S. (R.); Royal Red Cross, Second Class, Assistant Matron Louise Bennett, Sister Agnes Edgar, Sister Susanna Haig, Sister Emily Manser, Sister Helen Patterson, and Miss Jean Wells, Q.A.I.M.N.S. (R.); Sister Dean Lovibond, Egyptian A.M.N.S.; Matron Daisy Bulloch, Matron Mary Sinclair, and Sister Alice McConnon, B.R.C.S.; Miss Irene Anagnostopulo, Miss Lorna Cay, and Miss Alexandra Ross, V.A.D.

**ROUND THE HOSPITALS—(*continued*).**

Our readers, and particularly those who may have suffered disablement in consequence of their war services, will find much that is very important for them to know in the correspondence we publish this week between the Editor of The Hospital and Captain Murchie for the Director-General of Medical Services, Ministry of Pensions. Ever since the Armistice the demobilisation of nursing sisters has been in progress. Many thousands of them have scattered to every part of the kingdom, and to reach them collectively has been impossible. But it was and is imperatively necessary to bring to the notice of those who suffer in mind or body in consequence of their past exertions for the wounded information as to the right course to pursue in order to obtain relief. Every provision was made by an Army Order in August 1917 to ensure that nurses who were disabled should not be reduced to want. And so long as the Army nursing sisters remained on duty these provisions worked smoothly. Indeed, we understand that the number of nurses pensioned or relieved under this Army Order is not far short of 500. It is, however, notorious that the arrangements hitherto in force at the Ministry of Pensions for meeting demobilised nurses' disabilities and inquiring into their difficulties have been very far from efficient. Nurses have been very doubtful where they ought to go for help, and at the Ministry of Pensions, Westminster House, Millbank, their applications for help have not, to put it mildly, received great encouragement.

---

Under these circumstances we addressed a letter to the Ministry of Pensions, asking, in the interests of the nurses, for definite information as to the proper course for a nurse to pursue who is suffering in health as a consequence of her war work. Captain Murchie's reply is very courteous and concise. He tells us that the matter about which we are concerned, the difficulties of disabled nurses, is receiving attention, and that further information will soon be available. Meantime he states that individual nurses in need of care or treatment for disabilities due to or aggravated by war service ought to write to the Secretary, M.S. 2 (Officers), Ministry of Pensions, 14 Great Smith Street, Westminster, S.W. 1, and that their claims will be dealt with at once. It is necessary to enclose a letter or certificate from a medical man defining the ailment from which the applicant is suffering. Our readers will do good service if they will pass on the information thus afforded to any of their friends who may have suffered disablement and are uncertain how to press their claims.

---

It is satisfactory to learn that for the future the scope of the King's Fund for the Disabled has been extended so as to include disabled nurses who are in receipt of pensions under the Royal Warrant in respect of war service. Applications may be made to the Secretary, Millbank House, Millbank, S.W. 1. The main purpose is to assist nurses who are disabled from following their calling to start in other occupations or businesses, to change their home or proceed abroad either on medical grounds or to take up employment. The removal of this branch of aid from the jurisdiction of the Ministry of Pensions is an augury for good. The organiser who has in charge the nurses' department of the King's Fund will, we feel assured, answer letters promptly and arrange without delay for that personal interview without which no case ought ever to be decided. We believe that disabled nurses to whom neither pension nor gratuity has yet been awarded will shortly be assured equally practical and sympathetic aid in bringing their case to the ears of authority.

---

The West Kent General Hospital is fortunate in the inauguration of a Ladies' Association, which took place at a well-attended meeting at the hospital on October 6. Miss Furse, the matron, submitted a list of her requirements for the wards and patients, and Lady Samuel was elected President. It is hoped that many ladies who devoted their energies to making comforts for the wounded during the last five years will transfer their attention to the sick among the civilian population. It is certain that the new Ladies' Association will prove a source of additional strength to the hospital.

---

The British Committee of the French Red Cross is doing very fine work in the devastated regions of France, about which little information reaches this country. The problem with which France is faced is indeed gigantic. Crowds of refugees are returning to towns and villages which have been completely destroyed, and are struggling to get a home together again. In Montdidier, for instance, with a population of about 5,000 before the war, 700 have returned to the ruins. All are destitute and every one of them has received help from the British Committee. Tools, clothing, and furniture are the gifts most prized. The old people and children are provided with medical comforts, and a dispensary and surgery, with trained nurses, form part of the relief centre. Subscriptions may be sent to the London Committee of the French Red Cross, 9 Knightsbridge, S.W.1.

---

The Seventh Annual Conference of the National Association for the Prevention of Tuberculosis, which opened on October 16, was notable as the first held since the establishment of the Ministry of Health. Dr. Addison stated that the Ministry of Health, in conjunction with the Ministry of Pensions, were providing 1,000 training places in connection with sanatoria. They have definitely abandoned any idea of setting up any sort of tuberculosis colony, but settlements will be promoted in appropriate places. We trust the nursing sisters will not be overlooked in this far-reaching scheme. It is abundantly clear that there is not any adequate provision for them so far as regards training or sanatorium treatment, and there are many reasons why they should have sanatoria specially devoted to their requirements.

**ROUND THE HOSPITALS**—*(continued)*.

Queen Mary's Home, Hampstead, which will shortly be opened, has been fortunate in securing as matron Miss Wishart, who for nine years has been matron of the York Maternity Hospital. She leaves a large band of friends behind in the North, and is much regretted at the institution. No fewer than 125 applications were received in reply to an advertisement for a successor at the York Maternity Hospital, and the post was given to Miss Clara Elcoate, assistant county superintendent at Morpeth, Northumberland. Miss Elcoate's general training was taken at the Royal Victoria Infirmary, Newcastle-on-Tyne. She was superintendent of midwives at Manchester and had charge of the maternity department and midwifery pupils at the Jessop Hospital for Women at Sheffield. She served in the Army from August 1914 till last May, and her appointment is highly popular.

At examinations held in Exeter on October 10 and 11 by the Royal Sanitary Institute the following candidates received certificates:—Sanitary Science as applied to Buildings and Public works, E. B. Dewberry (one candidate failed); Maternity and Child Welfare Workers, none certificated (two candidates failed); Inspectors of Nuisances, Esther O. Broad, Arthur Gray, W. E. Hancock, W. T. Hawker, W. J. Luxton, Catherine W. Monaghan, William T. P. Pessell, Harold Settle, F. G. Vincent, (three failed); Women Health Visitors and School Nurses, Mary B. Hill (one candidate failed).

---

Few institutions did greater service for the wounded than the 2nd Western General Hospital at Whitworth Street, Manchester, which has recently been demobilised. It claimed, at the moment of its greatest activity, to be the largest hospital in the world. The Whitworth Street institution was a school till within a few days of its being prepared to receive the wounded. At its maximum the 2nd Western included 30 branch hospitals and 240 auxiliary hospitals, and numbered about 6,000 beds. Whitworth Street was the administrative centre of the hospital. The clerical and stores staff was very large and efficient and there was need that it should be, for in all some 270,000 patients passed through. Manchester has reason to be very proud of this gigantic organisation. The final sale which marks the break-up of the establishment has excited great interest and many are eager to secure, if only in the shape of a pot or a pan, a memento of its past glory.

---

For the future military hospitals in need of the services of V.A.D. members are instructed to apply to the Secretary of the War Office (A.M.O. 4), Cornwall House, Stamford Street, S.E.1., "through the usual official channels," and presumably it is here that V.A.D. members seeking employment, whether on first appointment or as reliefs for members who have left, should address their applications.

In 1916 the Home Secretary appointed a Juvenile Organisations Committee, under the jurisdiction of the Home Office. This useful committee has now been handed over to the Board of Education. The Right Hon. J. Herbert Lewis, Parliamentary Secretary to the Board, has become Chairman, while the former Chairman, Dr. A. H. Norris, remains a member of the committee. The Organising Secretary is Mr. C. E. Clift. The work demands the close co-operation of trained women workers, and, we trust, will receive it.

---

The American Red Cross relief unit, which has been working in Western Siberia for the past six months, reports a terrible epidemic of typhus among the Siberian troops. The fresh cases number 1,000 a day, and the total number this year has amounted to 130,000. All the resources of the hospitals for disinfection are exhausted and the epidemic is rapidly spreading among civilians and reaching enormous dimensions. All the cities are overcrowded with refugees from the Bolshevists, and near Omsk 30,000 persons are homeless with no resource but to dig shelters for themselves from the extreme cold in the ground. The mortality among the children is 30 per cent. Extensive assistance from the American Red Cross is the only hope of the starving population, for the country has been stripped of all medical and hospital supplies as well as of clothing.

---

The 1914 Star is now ready for issue to all units of the Army including the Military Nursing Services. Nurses who have not yet received their Stars should apply to the Secretary, War Office, (A. G. 10), 27 Pilgrim Street, E.C.4. The next-of-kin or legatees of deceased nurses may obtain the Star on application to the same address.

---

Lady Cowdray appeals for letters of admission to convalescent homes on behalf of nurses who have utterly broken down owing to the heavy strain of their work "under war conditions." It is most important that help should be afforded immediately to nurses who are worn out through their devotion to others, and the emergency is so great that only through a widespread response can every case of distress be dealt with. Presumably these are cases of home nurses overstrained in the task of carrying-on, of which there are innumerable instances. Everyone acquainted with nursing conditions understood the tremendous output of energy required to keep all the institutions for the sick going in England while 20,000 of the women usually engaged in this work were abroad. We are now reaping the harvest sown when holidays were relinquished, leave time curtailed, duty hours lengthened, and work intended for four pairs of hands was successfully carried through by one. Every nursing sister who can raise a convalescent letter among her friends should do so and send it to Lady Cowdray, British Women's Hospital Nation's Fund for Nurses, 32 North Audley Street, W. 1.

**ROUND THE HOSPITALS—(*continued*).**

The *Daily News* has opened its columns to a correspondence on "The Hard Lot of the Nurse," and a probationer writes from a large training-school in London dismayed at the prospect before her. She declares that one item in her timetable is alternate three months' night duty, " when one is in the wards for twelve hours on end; and of the other twelve hours only seven have been allotted to sleep." The condition has been to the discredit of those responsible, who have failed to realise the enormity of it. Improvement of the night nurses' lot marches very slowly indeed, and it sometimes seems as if nothing but an Act of Parliament restricting the hours will rescue nurses from the abnormal fatigue of the twelve hours' night. Another nurse, who argues that it stands to reason that a worn-out nurse cannot possibly give proper care to her patients, has justice on her side. We hear a good deal of the harm patients might get from having three relays of nurses instead of two in 24 hours. But is not the alterative far worse, to be tended by women who are too weary and nerve-strained to remember directions or enough awake to care what is happening in the sick-room?

---

At a meeting of the Medical Mission Auxiliary of the C.M.S., held at Leeds, Dr. Gordon Thompson gave a remarkable account of the work accomplished at his own mission station in the province of Yunnan, in the interior of China. Needless to say, the need for both doctors and nurses was emphatically brought to light. Some 25 per cent. of the male population are still slaves to opium in spite of the fact that the British Government have stopped the trade in the drug through the ports. It appears to be smuggled into China overland from Burmah.

---

At Bristol the local Voluntary Aid Detachment was demobilised on October 7 by Dr. Griffiths, the County Director, after "three minutes' preliminary," and some of the members think they might have received a word of thanks for their services before being told they might go home. But the fact is that the number of people who surrendered their time, their money, their health, their lives in the cause of their country is so vast that there are not enough thanks to go round. What is the good of going about trying to stroke the backs of all the people who did their duty? After all, we won the victory, and it did not always look as if that reward were going to be ours. What more do we want?

---

A clever article by Charlotte Aikens in the October number of the New York *Trained Nurse*, gives an able account of hospital activities in Argentina. In Buenos Aires there are 38 hospitals, British, French, German, Italian, and Spanish. The lazaretto, for infectious and incurable diseases, includes a leper colony, and is housed in magnificent grounds, with buildings covering 13 blocks connected by a miniature railway. The training of nurses is much hampered by lack of general education, the percentage of illiterates in the population being 72, in spite of a high-sounding educational system. Contrary to European tradition, the midwives, who receive two years' training and are registered, are of a higher type than the nurses, whose duties in the wards are approximately those performed here by ward-maids. In the British hospitals there is always a British nurse as matron, British sisters, and a few pupil nurses, but the increase of trained nurses in Argentina, year by year, is very small on account of the high marriage rate. English nurses, we learn, do not succeed well in native hospitals, much as they are needed there, as they are apt to be intolerant of native doctors. "Mrs. Evans, matron of the British Hospital (Buenos Aires), is one of those rare souls whom it is a privilege to meet."

---

The Walsall Guardians have been roused to a sense of their shortcomings by the report, presented by the Rev. H. O. Yeo, of a special committee appointed to inquire into the children housed among the workhouse and infirmary inmates. The report states that there are 30 children in the two institutions, some of whom have been inmates for years. They are receiving no education because most of them are unfit to attend the elementary school, and five are tuberculous. The committee recommended that they should be removed to other institutions. The Rev. H. McDonnell, in supporting the recommendation, declared that it was an altogether regrettable and undesirable state of things. Lack of energy in overcoming the difficulties imposed by war conditions is responsible for it, and the excuses made satisfy nobody.

---

An urgent appeal for help in combating typhus fever in Poland has been despatched by telegram by the League of Red Cross Societies from Geneva. The Medical Commission sent out to investigate the state of affairs at close quarters reports that to July 27 the number of cases declared was 124,000. The need for large quantities of soap, under and outer clothing, blankets, hospital equipment, utensils, and drugs, especially salvarsan, iodides, quinine, castor oil, salol, and opium, is urgent to the last degree. Many hospitals are without beds, sheets, or blankets. Fifty more doctors, and at least a hundred nurses, are wanted at once. The Commission urges the immediate necessity of obtaining doctors and nurses. At present the proportion of doctors is about one to every 10,000 inhabitants. Sir Arthur Stanley, presiding at the Conference on Tuberculosis on October 17, stated that he had that morning received the report of the Commission, and the recommendations made would go far to rescue the people of Poland from the awful epidemic. The International League of Red Cross Societies was showing in the first few months of its existence the value of co-ordinated effort.

